# Inflammatory Bowel Disease Associated Colorectal Neoplasia

**DOI:** 10.4172/2161-069X.S8-002

**Published:** 2012-01-23

**Authors:** Michelle Vu, Jyh-Yau Chang, Jeremy Chen, David Q. Shih

**Affiliations:** 1 Department of Medicine, Cedars-Sinai Medical Center, Los Angeles, CA, USA; 2 Inflammatory Bowel & Immunobiology Research Institute, Cedars-Sinai Medical Center, Los Angeles, CA 90048, USA

**Keywords:** IBD, Neoplasia, Ulcerative colitis, Crohn’s disease, Chemoprophylaxis

## Abstract

Patients with ulcerative colitis (UC) or Crohn’s colitis have a greater risk for developing colorectal cancer (CRC). Many studies have described the evolving epidemiology and risk factors for CRC in patients with inflammatory bowel disease (IBD). Recent evidence indicates that the incidence has been decreasing with the advancement of medical and surgical therapies, and surveillance has emerged as the foundation of prevention. Chemoprophylaxis is another area of research; however, given the limited efficacy of these agents, they are only being used in conjunction with endoscopic surveillance. Our ability to formulate effective strategies for the prevention of this dreaded complication expands as more is discovered of the molecular events underlying IBD carcinogenesis. Management strategies are constantly updated as new evidence and endoscopic techniques emerge. In this paper, we review the literature regarding epidemiology, pathogenesis, risk factors and chemoprophylaxis as well as the latest consensus guidelines for management of dysplasia and neoplasia in IBD patients.

## Introduction

Inflammatory bowel disease (IBD) carries an increased risk of developing colorectal cancer (CRC). Many molecular anomalies responsible for sporadic CRC are also seen in colitis-associated CRC, but differences in timing and frequency of these events indicate an alternate pathophysiology within these chronic inflammatory states. The risk of CRC increases with duration of colitis, extent of disease involvement, degree of histologic inflammation, family history of CRC, and concomitant primary sclerosing cholangitis (PSC). The decreasing incidence of this feared complication has been attributed to heightened surveillance and advances in medical therapy. Chemoprophylactic agents, including aminosalycilates (ASA), ursodeoxycholic acid (UDCA), and possibly folic acid, dampen the inflammatory milieu which predisposes to dysplasia. Colonoscopy with biopsies is the standard mode of dysplasia detection, and consensus guidelines afford structure to the surveillance schedule. In this article, we review the pathogenesis as well as current consensus guidelines for screening and surveillance of CRC in IBD.

## Epidemiology

The increased risk of CRC in IBD has been established for decades. Varying data has compounded the challenge of quantifying this risk. Differences in study design, length of follow-up, case definitions, environmental diversity, treatment strategies and referral center bias contribute to the wide range of risk seen in the literature.

The reported risk of developing CRC in the background of ulcerative colitis (UC) ranges from no increased probability versus the general population to a 34% chance of developing CRC after 25 years disease duration [1–8]. Initial studies from tertiary referral centers often overestimated the CRC risk in IBD, as patients from these institutions often present with more severe illness or later in the disease course [2–6]. Because of potential miscalculation and variation of CRC risk in hospital-based populations, there was a subsequent shift of focus toward population-based studies that potentially afford greater external validity. These results were more conservative and demonstrated CRC rates that approached that of the general population. A 2001 meta-analysis attempted to sort through the data in 116 studies and found the overall prevalence of CRC in UC patients to be 3.7% (95% confidence interval (CI) 3.2–4.2), with an increase to 5.4% in patients with pancolitis. The cumulative risk of developing CRC in any UC patient was estimated at 2% at 10 years of disease duration, 8% at 20 years, and 18% at 30 years [9].

An association between Crohn’s disease (CD) and CRC has been suggested, with results ranging from no increase in risk to a 20 times increased risk [3,8,10]. The first long-term study investigating CRC risk in CD followed 449 patients with CD symptoms prior to 21 years of age. All cases of CRC occurred in patients with colonic disease, and the risk of CRC was estimated to be 20 times greater than in the general population [3]. A later hospital-based study examining CRC risk in CD patients with an intact colon showed relative risk (RR) increase of 4.3, with RR increasing further to 23.8 when patients with extensive colonic disease were examined [10]. A 2006 meta-analysis found that in six studies examining the difference of CRC risk in CD patients versus general population, the overall RR for developing CRC in CD patients was 2.5 (95% CI, 1.3–4.7, p=0.004). In patients with Crohn’s colitis, the RR was greater at 4.5 (95% CI, 1.3–14.9, p<0.015). Sub-analysis based on referral location (primary, secondary or tertiary center) showed no difference in risk. The cumulative risk of developing CRC in CD at any site was calculated to be 2.9% at 10 years of disease duration, 5.6% at 20 years and 8.3% at 30 years [11].

Later studies examined the risk of CRC in both UC and CD patients, and found less drastic numbers but a persistently increased incidence of CRC in the IBD patient population. A 2001 study of CRC in IBD patients showed similar increase in RR for CRC in UC (colon cancer 2.75; 95% CI, 1.91–3.97; rectal cancer 2.64; 95% CI, 1.69–4.12) and CD (CRC 2.64; 95% CI, 1.69–4.12) [12]. A 2006 population-based study of patients in Olmsted County, Minnesota showed no increase in risk for CRC in any UC patients with a standardized incidence ratio (SIR) 1.1 (95% CI, 0.4–2.4), but a secondary analysis demonstrated a greater CRC risk in the setting of extensive colitis with a SIR 2.4 (95% CI, 0.6–6.0). The cumulative incidence of CRC in UC patients was estimated to be at 2.0% at 25 years after diagnosis. Based on extent of disease, the CRC risk was lowest in proctitis patients (SIR 0.0; 95% CI, 0.0–3.5) and greatest in patients with extensive colitis (SIR 2.4; 95% CI, 0.6–6.0) [13]. This decreasing incidence of CRC over the past few decades is likely a testament to the success of constant surveillance and more successful control over disease activity with the advent of new therapeutics.

## Molecular Events

The pathogenesis of IBD involves immune system dysregulation in response to gut flora in a genetically predisposed individual, leading to a state of chronic intestinal inflammation [14]. Normal immune activation triggers a tumor suppressive response, but it is believed that this security system fails in the face of chronic inflammation. The altered immune response allows for proliferation of dysplastic cells, which are stimulated by proinflammatory cytokines and growth factors [15,16].

UC and CD are driven by different T helper cell mediated immune responses, which result in these two distinct phenotypes. Duration and degree of intestinal inflammation is a risk factor for CRC in IBD, and the immune system and cytokines that mediate inflammation have been investigated regarding disease pathogenesis. While UC consists of a Th2 response with high IL-5 and IL-13, CD is primarily a Th1 response with elevated TNFα and IFNγ [17]. In models of sporadic CRC, a Th2 response is associated with further disease progression, while a Th1 response is considered protective [18,19]. Recent research has implicated a coexisting Th17 response in CD as the reason for the higher CRC risk seen in observed in CD patients [20,21]. The mechanisms underlying these distinct conditions and implications on the development of colorectal neoplasia have yet to be fully elucidated.

The contribution of innate immune activity in IBD carcinogenesis is further suggested by recent research implicating epithelial toll-like receptor 4 (TLR4) as a potential marker for dysplasia and target for preventing CRC in colitis [22]. Transgenic murine models of colitis with elevated epithelial TLR4 developed severe acute inflammation, greater colitis-associated neoplasia, and elevated levels of inflammatory and pro-oncogenic factors (TNFα, COX-2, PGE-2) versus wild type (WT) mice [23,24]. When the epithelial TLR4 signaling pathway was blocked in WT mice with colitis-associated tumors, there were fewer colonic polyps. Furthermore, in UC patients with associated dysplasia or neoplasia, the level of TLR4 expression increased with dysplastic grade [22].

While sporadic CRC typically sprouts from mucosal polyps, morphologic changes in colitis-associated CRC are heterogeneous and may present as flat or raised lesions. Despite the challenge of identifying these changes in a chronically inflamed colon, detection of these changes is imperative as IBD patients carry a higher risk of developing CRC. Sporadic CRC arises because of genomic instability and the two major types, chromosomal instability (CSI) and microsatellite instability (MSI), account for approximately 85% and 15% of sporadic CRC respectively. While these mechanisms are also observed in colitis-associated CRC, the timing and frequency is different in IBD [25].

Chromosomal instability, a function of abnormal chromosomal segregation and DNA aneuploidy, is the reason for most p53 and APC dysfunction. APC dysfunction is typically an early event in the adenoma-carcinoma pathway of sporadic CRC, while p53 mutations take place at later stages of disease. In colitis-associated CRC, this is reversed- APC defects are less frequent and seen later in disease course, whereas p53 chromosomal abnormalities observed in earlier stages [26–28]. This finding may account for the flat morphology of dysplasia observed in IBD-associated CRC, as APC mutations are considered the reason for polyp formation. Errors in the p53 tumor suppressor gene, which is contained within chromosome 17p, occur earlier and more frequently even in nondysplastic mucosa, suggesting that underlying CSIs provide the background for CRC development in IBD patients [29]. The molecular events behind p53 dysfunction have been explored in animal models of colitis. One proposed mechanism is that matrix metalloproteinase-9 (MMP-9) has a tumor suppressive effect through Notch1 and subsequent p53 activation. Notch1 signaling in sporadic colon cancer induces adenoma formation in animal models [30–32] whereas studies have found a protective role against tumor formation in colitis-an effect theorized attributed to its inflamed environment [33]. Murine models deficient in MMP-9, which activates the transcription factor Notch1, have increased susceptibility to CRC [34]. The downstream effects of MMP-9 were elucidated when Notch1 inhibition led to lower levels of p53 and higher levels of tumor in and dysplasia compared to control mice [33]. MSI involves failure of the DNA mismatch repair system, which is comprised of proteins that coordinate DNA repair. In sporadic CRC, transcriptional silencing of the hMLH1 promoter via methylation accounts for a major part of MSI CRCs [35,36]. IBD patients with high levels of DNA MSI more often have heterogenous defects in hMLH1, hMSH2, hMSH6 and hPMS2 mismatch repair genes, and less frequently had hMLH1 promoter methylation [37]. Chronic inflammation may predispose the colorectal mucosa of IBD patients to genomic instability, thus accounting for the progression of the genetic changes of colitis-associated CRC. Unlike sporadic CRC, dysplasia and neoplasia in IBD patients typically arise from many discrete areas of a colitic environment.

Biologic markers have been explored for their role in carcinogenesis and potential value in identifying dysplasia in an inflammatory milieu. Chitinase 3-like-1 (CHI3L1) is one of these factors expressed in colonic tumors [38,39], and levels of CHI3L1 mRNA are also greater in patients with active IBD [38,40,41]. Specifically, CHI3L1 is elevated only in colonic epithelium of colitic areas and this has prompted investigations into the role of CHI3L1 in IBD carcinogenesis. UC patients with remote dysplasia were found to have significantly higher CHI3L1 mRNA expression than UC patients without remote dysplasia (p=0.05) [42]. When compared to normal individuals, its expression was 20 times greater in UC patients with remote dysplasia (p >0.001). These results have implications for the future use of this biomarker in distinguishing IBD patients at greatest risk for malignancy. Aldehyde dehydrogenase isoform 1 (ALDH1) has been established as a colonic stem cell marker, and is upregulated in colonic stem cells [43]. In cancer stem cells, ALDH1 is similarly active and protects against cell destruction [43]. Staining for ALDH1 to distinguish inflammatory from premalignant changes yielded a high sensitivity (92% for CRC or HGD, 95% for LGD), but poor specificity (55% for dysplasia) as many inflammatory changes were also ALDH1 positive [44]. The presence of high ALDH1 implicates colonic stem cells in the spectrum of processes, including inflammation and dysplasia, in IBD carcinogenesis.

## Risk Factors

Association of an increased CRC risk in the background of IBD has been described extensively in the setting of UC, and more recently has been demonstrated in certain CD patients. Environmental, genetic and individual factors synergistically increase the risk of colorectal dysplasia and neoplasia in these patients. Summary of the IBD associated CRC risk factors are listed in Table 1.

Cumulative cancer risk increases with age in the general population; in IBD patients, this hazard is further compounded by specific disease characteristics including disease duration, area of colonic involvement, and degree of inflammation. A longer duration of colorectal inflammation has consistently been correlated with higher rates of CRC in IBD patients, although this rate has decreased with the advent of surveillance colonoscopy [9,11,45–47]. Extent of disease is another independent risk factor for development of CRC. A study of Swedish UC patients demonstrated a RR of CRC of 1.7 in ulcerative proctitis, 2.8 in left-sided colitis, and 14.8 in extensive colitis [8]. The 19-fold increased risk of CRC in patients with extensive UC was reproduced in patients with extensive Crohn’s colitis, who demonstrated a 20-fold excess risk [2]. A few recent studies have shown that greater degree of histologic inflammation, when controlling for duration and extent of disease, also predicts higher risk of CRC in UC patients [48,49]. The presence of backwash ileitis (BWI) in UC patients with pancolitis has been investigated for its predictive value for CRC [50]. Ulcerative pancolitis patients with BWI had a greater odds ratio (OR) of CRC than those without BWI (OR 19.36 vs. 9.58, p<0.001), although a confounding association of BWI with primary sclerosing cholangitis (PSC) was also noted [51,52]. Interestingly, higher disease activity as indicated by more frequent clinical exacerbations was not found to be a risk factor for development of CRC [53].

Other studies have shown that CRC as a complication of IBD is greater in patients with an earlier age at IBD diagnosis, regardless of duration of colitis. Crohn’s patients with colonic involvement diagnosed at a younger age had higher rates of CRC [54,55]. In a Swedish population, the RR of CRC was 20.9 in Crohn’s colitis patients diagnosed before 30 years old versus those diagnosed at later ages (RR 2.2) [54]. In this same population, UC patients with pancolitis diagnosed prior to 15 years old had an absolute CRC risk of 40 percent after 35 years of disease, versus 30 percent in all age groups [8]. The results of this population-based study are similar to a referral center study of CRC in UC patients diagnosed prior to 15 years old which yielded a cumulative incidence of 43 percent by 35 years disease duration.

A positive family history of CRC, regardless of family history of IBD, is independently associated with increased CRC risk [56,57]. One Mayo clinic study showed that UC patients with a first-degree relative of CRC had a 2 times greater risk of developing CRC, irrespective of extent and duration of colitis [57]. The presence of PSC further adds to the CRC risk in IBD patients [51,52,58,59]. A meta-analysis of eleven studies showed that UC patients with concomitant PSC have an increased risk of CRC, with an OR of 4.09 (95% CI, 2.89–5.76) versus UC patients without PSC [60]. In these higher-risk groups, earlier screening and aggressive surveillance guidelines have been instituted to mitigate this hazard and, if necessary, perform colectomy.

Smoking tobacco has been shown to exacerbate CD, but it exerts a protective effect in UC [61–63]. The increased risk of CRC in cigarette smokers and the positive effect of cigarette smoking on the disease course of UC have been well-established. Animal models of colitis that were exposed to cigarette smoke had a greater rate of colonic adenoma and adenocarcinoma [64]; however, the degree to which cigarette smoking in UC patients alters molecular events in IBD carcinogenesis is still unknown. A subgroup analysis in a case-control study of UC patients did reveal an association with smoking and a lower risk of CRC versus nonsmokers, but further investigation is warranted [65].

## Surveillance

**Figure1:**
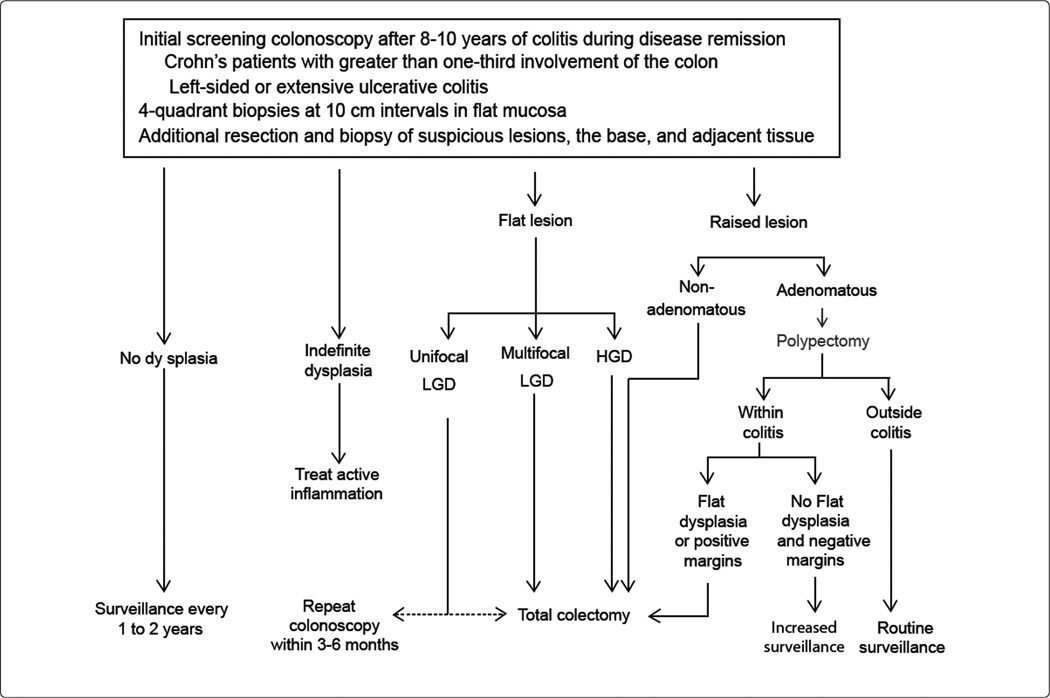
Summary of IBD colorectal neoplasia surveillance. LGD (low grade dysplasia), HGD (high grade dysplasia).

Dysplasia is a known precursor to CRC and is defined as epithelial neoplasia confined to the basement membrane [66]. The goal of surveillance is to identify these premalignant changes and allow for intervention that will forestall the development of CRC and its dreaded complications. Regular colonoscopy is the mainstay of surveillance. In the general population, premalignant lesions present as prominent colonic polyps which can be resected during colonoscopy, and these patients are thereafter subjected to more rigorous surveillance. Detection of colorectal dysplasia in high-risk IBD patients presents an even greater challenge. Figure 1 outlines the current surveillance guideline for IBD patients.

The macroscopic appearance of dysplasia in IBD patients is classified as flat or raised. Flat dysplasia can occur within areas of normal colonic mucosa, and newer methods have been aimed at detection of this elusive entity. In addition to flat dysplasia, dysplasia can be found in plaques and lesions (dysplasia associated lesion or mass, DALMs) or resemble sporadic adenomas surrounded by normal appearing colonic mucosa (adenoma-like lesion or mass, ALMs). While ALMs have low malignant potential and may be removed via polypectomy, the presence of DALM is associated with concomitant CRC [67–69]. In a review of 10 surveillance programs, CRC was found in 43% of colectomies performed because of a DALM with any dysplasia grade [70].

Dysplasia is classified histologically as indefinite, low-grade and high-grade [66]. While this system is commonly used, it is subject to poor interobserver agreement in histopathologic interpretation [71]. One study found only 60% agreement in diagnosing LGD [72], although very high levels of agreement has been observed in negative specimens or in HGD (kappa statistic for gastrointestinal pathologists 0.30, 95% CI, 0.26–0.34; for general pathologists 0.28, 95% CI, 0.23–0.32) [73].

In UC patients with dysplasia who underwent colectomy, CRC was found in 19% of patients with LGD and 42% of patients with HGD [70]. Further analysis revealed that 29% of patients with LGD had eventual progression to HGD, DALM or CRC. Some evidence indicates that flat LGD in particular has a tendency to transform into HGD or cancer, with the rate of neoplastic transformation of flat LGD being 53% after 5 years [74–76]. A meta-analysis of twenty surveillance studies found that LGD carried a 9 times increased risk of developing CRC (OR 9.0, 95% 4.0–20.5), and a 12 times increased risk of developing any advanced lesion (OR 11.9, 95% CI, 5.2–27) [77]. The association with dysplasia and colitis secondary to CD also holds true. Small series of CD patients with CRC report similarly high rates of concomitant high-grade dysplasia [78,79]. General consensus is that whenever DALM or HGD is identified, a colectomy should be strongly recommended [70]. Patients with LGD should likewise be considered for colectomy given the risk of malignant transformation [80].

Although colonoscopy is widely accepted as standard method to detect dysplasia and neoplasia in IBD, it has several limitations. These include incomplete patient and practitioner adherence, invasiveness, high cost, endoscopic sampling variations and poor interobserver agreement of histologic analysis. The major question is whether or not surveillance actually has an impact on mortality rates. A Cochrane review of three studies concluded that there was no concrete evidence for improved survival in patients with extensive colitis (RR 0.81, 95% CI, 0.17–3.83), although there was a trend toward earlier stage of CRC diagnosis which has been associated with improved outcomes [81].

Surveillance should begin 8–10 years after symptom onset in pancolitis, and should be done annually or biannually with 2–4 random biopsies at 10 cm intervals and additional samples from suspicious areas.

Despite the known potential for conversion of dysplasia to neoplasia and the possibility of surveillance failure, many patients opt for surveillance over prophylactic colectomy with the understanding that they are at increased risk for cancer and warrant heightened surveillance. This is not unreasonable, as dysplasia can potentially regress or remain stable over time [82]. Because the risk of CRC in Crohn’s colitis is comparable to UC, guidelines are similar for both processes. Surveillance colonoscopy should be initiated 8–10 years after symptom onset in pancolitis and while disease is in remission, as active inflammation may obscure the distinction between reactive atypia and dysplasia [83,84]. One study calculated that in order to achieve 90% confidence of dysplasia detection, 33 random biopsies should be obtained [85]. To achieve 95% confidence of dysplasia detection, the authors found that 64 random biopsy specimens are required. The 2005 Crohn’s and Colitis Foundation of America (CCFA) Consensus advocates four-quadrant biopsies at 10 cm intervals, with additional samples in suspicious areas or in the lower sigmoid and rectum [86]. The CCFA recommends surveillance every 1 to 2 years in patients with extensive or left-sided UC and patients with Crohn’s colitis with one third colonic involvement. After two negative colonoscopies, surveillance should occur every 1 to 3 years, and then every 1 to 2 years once the disease has been present for 20 years [86]. There are no strict guidelines stating the optimal surveillance interval, but because the risk of cancer increases with time, the frequency of screening should likewise increase with each decade of disease [87,88].

Recommendations have been further refined in UC patients. Studies in surveillance programs have uncovered a predilection for distal neoplasa, with 47–68% of CRC occurring in the rectosigmoid region [80,89]. In a recent 2011 study, Goldstone et al noted that in 103 patients with extensive colitis, neoplasia occurred in the rectosigmoid colon significantly more than other regions (*p*<0.0007). This also remained true in subgroup analyses of patients with advanced and flat neoplasia [87]. Patients with PSC should undergo surveillance at the time of diagnosis and annually thereafter [86].

## Prophylaxis

The mainstay of chemoprophylaxis is the attenuation of CRC risk by identifying and treating reversible risk factors. Some of these factors that play a role in development of dysplasia and neoplasia include chronic inflammation, vitamin deficiencies and environmental exposures. Chemoprevention has been investigated as an adjunct to surveillance, as even optimal surveillance does not afford complete protection against development of CRC.

Chronic, uncontrolled inflammation is a factor in malignant transformation, and anti-inflammatory medications have been investigated for a possible role in primary chemopropylaxis. Aminosalicylates (ASA) block the NFκB pathway which results in apoptosis and reduction of tumor [90]. Nonsteroidal anti-inflammatory drugs (NSAIDs) decrease the risk of sporadic CRC by induction of apoptosis and inhibition of colonic epithelial growth in sporadic polyps. These compounds have been assessed in the setting of IBD for a protective effect against CRC. While there have been no prospective trials evaluating the beneficial effect of ASA on CRC in IBD patients, these compounds have long been used for their therapeutic and potential chemoprophylactic effects. A case control study of 102 cases of CRC in UC patients showed a 75% decreased risk of CRC in patients who used any ASA compounds [53]. The greatest protective effect against CRC was seen with mesalamine at doses greater than 1.2 grams/day (OR 0.09, 95% CI, 0.03–0.28). A 2005 meta-analysis of 9 studies demonstrated a protective association with the regular use of 5-ASA and dysplasia or CRC (OR 0.51, 95% CI, 0.38–0.69) [91].

Other therapeutics with anti-inflammatory properties has been evaluated for a chemopreventive effect, without much success. While systemic and topical steroids are associated with reduction in CRC risk, their long-term use for chemoprophylaxis is not feasible given the extensive side effect profile [53,92]. Biologics such as azathioprine and 6-mercaptopurine do not appear to have any role in reduction of CRC risk [93].

Folate deficiency has been implicated in development of CRC via induction of DNA strand breaks in the *p53* tumor suppressor gene [94]. One small study found red blood cell folate levels, which reflect body stores, to be significantly lower in UC patients with CRC versus patients without CRC [95]. The CRC risk has been reported to be as high as 17 times greater in IBD patients with hyperhomocysteinemia and folate deficiency [96]. A retrospective study did reveal a trend for dose-dependent protective effect of folate supplementation against neoplasia in UC [97], but a significant effect of folate supplementation on CRC development has yet to be reliably reproduced. While folate is not considered an effective chemoprophylactic agent, clinicians will typically correct this deficiency in IBD patients.

Patients with PSC and IBD may benefit from ursodeoxycholic acid, which decreases colonic exposure to carcinogenic bile acids. A retrospective cross-sectional study of patients with PSC and UC showed that regular use of UDCA was associated with a decreased risk of colonic dysplasia (OR 0.18, 95% CI, 0.05–0.61). This was reproduced in a prospective randomized placebo-controlled trial in which patients with PSC and UC who were taking UDCA had a RR of 0.26 (95% CI, 0.06–0.92) for developing CRC [98].

Undergoing a total colectomy would obviate the need for surveillance as it completely eliminates the chance of CRC. The efficacy of a total colectomy in prevention of CRC can be inferred by from a 2006 population-based study from Copenhagen which reported a 0.06% annual risk of CRC in UC patients, and a 30-year cumulative CRC risk of only 2.1%. The authors of this study attributed their low results to use of 5-ASA maintenance therapy in 70% of patients and an aggressive surgical approach to treatment in their population [1]. In patients with a single, flat low-grade dysplastic lesion on their first surveillance colonoscopy, immediate colectomy has been found to be preferable in terms of costs-effectiveness and quality-adjusted-life-years ratios versus continued surveillance [99].

## Conclusion

CRC risk is increased in IBD patients. The risk is related to the duration and anatomic extent of the disease. Many molecular anomalies responsible for sporadic CRC are also seen in colitis-associated CRC, but differences in timing and frequency of these events indicate an alternate pathophysiology within these chronic inflammatory states. Although no large controlled trials have proven that surveillance reduces mortality, surveillance is widely practiced and recommended by major gastroenterology societies.

## Figures and Tables

**Table 1:** IBD Associated Colorectal Neoplasia Risk Factors.

Risk Factors	Reference	IBD	Results
Extensive Colitis	[8]	UC	Relative risk of neoplasia RR 1.7 (95% CI, 0.8–3.2) in ulcerative proctitis RR 2.8 (95% CI, 1.6–4.4) in left-sided colitis RR 14.8 (95% CI, 11.4–18.9) in extensive colitis
	[53]	UC, CD	Relative risk of neoplasia RR 19.2, (95% CI, 12.9–27.5) p<0.001 in UC RR 18.2, (95% CI, 7.8–35.8) p<0.001 in Crohn’ colitis
Histological Inflammation	[46]	UC	OR 6.98 (95% CI, 2.42–20.16) p<0.001 for neoplasia
	[47]	UC	HR 1.4 (95% CI, 0.9–2.3) for any neoplasia HR 3.0 (95% CI, 1.4–6.3) for advanced neoplasia
Backwash Ileitis	[48]	UC	OR 19.36 (95% CI, 4.6–135.8) p<0.001 for neoplasia
Age at Diagnosis	[8]	CD	SIR 12.025 (95% CI, 3.1–23.2) for neoplasia with IBD diagnosis <30 yo
	[8]	UC	SIR 118.3 (95% CI, 63.0–202.3) for neoplasia with UC diagnosis <15 yo SIR 16.5 (95% CI, 10.2–25.2) for neoplasia with UC diagnosis 15–29 yo
CRC Family History	[54]	UC, CD	Relative risk of neoplasia RR 2.0 (95% CI, 1.0–4.1) in UC RR 3.7 (95% CI, 1.4–9.4) in CD
	[55]	UC	OR 2.33 (95% CI, 1.06–5.14) p=0.03 for neoplasia
Concomitant PSC	[49]	UC	Adjusted RR 3.15 (95% CI, 1.37–7.27) for neoplasia or dysplasia
	[50]	UC	Adjusted RR 10.4 (95% CI, 4.1–26.1) for neoplasia or dysplasia

RR=relative risk, OR= odds ratio, HR= hazard ratio, SIR= standard incidence ratio, CI= confidence interval

## Abbreviations

ALMs: Adenoma-like Lesion or Mass
ALDH1: Aldehyde Dehydrogenase isoform 1
ASA: Aminosalicylates
CHI3L1: Chitinase 3-Like-1
CRC: Colorectal Cancer
CCFA: Crohn’s and Colitis Foundation Of America
CD: Crohn’s Disease
DALMs: Dysplasia Associated Lesion or Mass
IBD: Inflammatory Bowel Disease
MMP-9: Matrix Metalloproteinase-9
NSAIDs: Non-Steroidal Anti-Inflammatory Drugs
PSC: Primary Sclerosing Cholangitis
TLR4: Toll-Like Receptor 4
UC: Ulcerative Colitis
UDCA: Ursodeoxycholic Acid
WT: Wild Type
